# Extra Supplement—The Nursing Mirror

**Published:** 1889-03-09

**Authors:** 


### The Nursing Mirror.

All Contributions for this Supplement should be addressed "The Nursing Mirror," care of The Editor, 38, Old Jewry, E.C.

### En Passant.

SCARBOROUGH SISTERS.—It is not so well known as it ought to be that Miss Edith Williamson has started a nurses' home at Scarborough. Miss Williamson is a sister of Sister Dora—who is a parish nurse in Scarborough. The great advantage of the home is that nurses are sent out for any length of time, for an hour, for a night, or for a week.

STAMFORD NURSING SOCIETY.—We have received the annual report of this society, which is ssued in neat grey cover, and occupies many pages. We were rather amused to find after much searching that this book contained only the account of one nurse's work. Of course it is right to place all these matters on a thoroughly businesslike basis, yet the accounts and reports of a parish nurse can generally be cheaply yet fully printed on a sheet of paper. However, we congratulate Nurse Blackmore on having such a delightful publication all to herself.

REST AND RELAXATION.—For those nurses who can at times secure a little freedom and like to make the best use of it, we would mention that the last Ballad Concerts take place on March 13th and 20th. During Friday evenings in Lent Bach's Passion music will be sung at St. Anne's Church, Soho ; tickets can be had by sending stamped directed envelope to Canon Wade, The Rectory, Soho Square. On Wednesday evenings at eight during Lent Dr. Vaughan will preach at the Temple Church, and vouchers will not be necessary to secure admission. For those who are more frivolously inclined we may mention that "Sweet Lavender" at Terry's Theatre is very amusing, and touches on nursing subjects, introducing a very pretty sister. The first piece at the Vaudeville also contains an amusing dialogue anent a hospital, and "That Doctor Cupid" is full of fun and laughter. Every Saturday afternoon at three p.m. there are lectures in the South Kensington Museum Lecture Hall of an excellent nature ; admission 1s. Professor Lockyer began the course on February 16th, and now Mr. Starkie Gardner is delivering two lectures on "Iron."

THE PHYSICAL STRAIN OF NURSING.—In a letter to the *Standard* last week a doctor wrote a word of warning to the young girls who now besiege the hospitals to secure situations as probationers. He said : "From a home of probably comparative ease, where their hardest day's physical labour is a morning spent in light house work, followed by an afternoon's lawn tennis, these scarcely-formed, tender women enter upon the stern duties of a nurse. From eight a.m.—in some cases it is from seven a.m.—until eight p.m., or even later, with but short intervals for meals, they are 'on duty.' Do any of those who have not tried it know what that 'on duty' means. I fear not. It is literally on foot for the whole time, with the addition of lifting patients, heavy with helplessness and pain, bending over beds, mounting stairs—in fact, everything tedious and toilsome. Such a change from the life that most of these girls have been accustomed to lead has, as a rule, but one ending, and that is a breakdown in health. Much has been done for nursing by the sacrifices of noble women ; but surely tender girls, wholly unaccustomed to such physical strain as that put upon them in our hospital wards, ought not to be allowed to blindly ruin themselves in health and happiness. There was much stir made some years ago about the girls standing in our shops ; there is more need now for a similar outcry against the strain upon our nurses and nurse probationers. Girls brought up to domestic service can stand this strain, and make excellent nurses ; ladies, accustomed only to play at work, simply ruin themselves, and bring trouble and misery upon their homes. I speak as one having authority, for I have seen these evils brought only too close to my own hearth. Parents and guardians, brothers and lovers, take warning in time, or our Women's Hospitals will soon be full of those who, through misguided zeal, went forth to nurse and returned home to be nursed for the best part, if not for the rest, of their lives."

"WORK AND LEISURE."—The magazine which is published under the above title always contains much that is of interest to all women, besides items of special interest to nurses. In the March number there is a capital article on "The Training of the Brain," which though chiefly addressed to mothers is yet applicable to all who aim at self-improvement. Then there is a sensible account of laundry work for ladies, pointing out the advantages and disadvantages of this employment. A very appreciative notice of *Nursing Notes* also finds a place in this number, and an account of that charming health resort, Buxton, is well worth reading.

ANXIOUS NURSES.—We have been much amused at some letters lately received from nurses in the provinces. A widespread fear seems to have arisen—we do not care to inquire how—that the registration of nurses may be established any day, like a thunderclap, and that then those nurses who do not belong to the magical society which advocates registration will be left without the means of earning their living. Such a notion is of course utterly erroneous and absurd ; if a general registration ever does come it will probably be after this generation has passed away ; Bills generally spend many years in passing through Parliament, and many more in being prepared for that ordeal. Look at the question of the registration of midwives ; it has been a burning subject and widely advocated for at least a dozen years, and only now does the faintest hope of its becoming law begin to dawn on the minds of its promoters. If nurses are paying yearly half-crowns with any thought of securing registration for themselves, and not for their grandchildren, we are sorry for them ; but we feel genuine concern for those nurses who, through fear, are yearly wasting their money. If registration ever comes, it will not be through the hands of a clique, but through the law of England, and will have some regard for justice. If it comes it will come to all, whether they have paid half-a-crown a-year or not. Our anxious friends in the provinces may banish their fears for the present ; perhaps twenty years hence they may require them again, or perhaps by that time the very name of the society they dread will be utterly forgotten.

HEROISM.—Mr. Oakeley Archer has written to the Melbourne *Argus* on the subject of the fire at the Alfred Hospital, of which we gave an account last week. Mr. Archer seems to have been much struck with the behaviour of the nurses, and to have thought it worthy of public recognition. He therefore sends the following letter to the *Argus*, together with a cheque for three guineas : "It was a sight never to be forgotten ; ladies carrying great helpless men on mattresses, staggering under their weight, and shielding them with sheets from the burning *debris* that kept falling on them from the roof ; and all the time as bright and cheerful as if nothing was the matter. One false step, one moment's loss of self-command, and the result would have been appalling. Thirty helpless men would have been roasted in their beds. But no, they worked like heroes, and not only saved all their lives, but did their work in such a careful manner that not a single patient was a bit the worse for being moved. I don't want to say that these women did more than their duty, but I certainly think that they deserve some public recognition for their bravery. During the fire two of them lost all their personal effects, their room being burnt while they were attending to their patients in the execution of their duty. I am sure all my old fellow-patients, remembering the kindness and attention we received, and which we all know can never be repaid, will join me in procuring a suitable *souvenir* for each and every one, from the matron to the youngest pupil, and I ask you to receive subscriptions as well as give publicity to this matter."

### Nurse Eyre's Mistake.

"Who are you?"

It was a quaintly-furnished little room at the back of a large, old Queen Anne house set in the midst of a fine open country. In an antique carved chair sat the Master of Chudleigh, and on a stool rested his gouty foot, the torment of the old soldier's life. Of late the enemy had taken a still more violent hold, and, in consequence, the meek, long-suffering country doctor had plucked up the courage to insist upon a couple of nurses being sent for from town.

"If you don't, Sir John," said he mildly, "you'll lay up every man and woman in the house. You know you've constantly got a string of them pottering about you, whereas, if we get down two good nurses from St. Ruth's, things will be put on a methodical footing at once."

"Confound you, sir!" roared the amiable patient; "do you think you're going to see me tied to the apron strings of a couple of conceited women? Oh-h!" A deadly twinge converted the stentorian roar into a helpless whine. "But there, do as you like—do as you like; only don't send for two—have some mercy, get one, and let her be middle-aged, for then, perhaps, she won't think she knows quite so much."

The days went by, and Sir John grew worse. Between his agonies and his furies the servants were terrified to enter the room, and the Master of Chudleigh had neither wife nor child to stand in the breach. So things had come to a melancholy pass, when, on a certain afternoon, Sir John, lifting his eyes, caught sight of a little figure standing framed in the doorway, quietly taking stock of himself and his surroundings.

"Who are you?" The repeated question was well nigh shouted.

"I'm Nurse Eyre, Sir John." It was a sweet voice that fell on the air, still vibrating with the stentorian accents of the invalid. "I've just arrived." And the little figure came close up to the carved chair. "I've had a long journey, but they gave me some hot tea, and I feel quite revived." The cheery voice ceased, and Sir John was speechless—dumb with surprise. What was the world coming to? Felt revived, did she? What did he care!

"What d'ye mean, ma'am, by not being middle-aged?" he growled at last, his eyes glittering under the shaggy brows.

"I didn't know you wanted me to be middle-aged; but I'm twenty-four, so I'm getting on, and you needn't say ma'am'; I'm usually called Nurse Eyre," and the tranquil speaker, drawing a low chair up, seated herself, and began to talk about her journey.

She was a small creature, with large grey eyes and long black lashes—Irish eyes. Her fair hair was smoothly folded round the erect little head, and the invalid's frown grew slightly mollified at the rare spectacle. "As smooth as the women's heads used to be in my young days," he muttered.

But the most astonishing thing about the stranger was her confidingness; she seemed to take it for granted that Sir John was hugely pleased to see her, and her cheery chatter went on uninterruptedly.

"Well, I never did!" said Grimshaw, the butler, as he deposited his comfortable person at the well-spread tea-table in the housekeeper's room. "Man and boy I've lived with Sir John this thirty year, and I never see anyone speak up to 'im like that London nurse. It fairly took my breath away—a bit of a thing not much higher than the table."

"Yes but, Mr. Grimshaw," put in portly Mrs. Barnes, "I've heard tell that they are real born ladies some of them nurses in London town, though in my young days it were different; and as for this new-comer, you can't deceive me—I know the marks of birth and breeding, and she's got 'em. She's somebody, there's no manner of doubt; perhaps a title reduced," and Mrs. Barnes glanced longingly at her unopened budget of weekly fiction, just laid upon the table.

"Well, whoever she is, she's makin' a grand mistake. Why, she's behavin' as if the guv'nor were delighted to see her, and looked upon her as a honoured guest. Yes, she's makin' a mistake."

But Grimshaw was wrong. Nurse Eyre was not making a mistake; or, if she were, a mistake meant a miracle. She had not been a week in the house before Sir John's tyrannical voice became almost gentle. His hapless limb tended by a skilled hand, his bandages perfectly adjusted, his little needs forestalled, all smoothed away his fractiousness, and caused his softened eyes to rest contentedly upon the sunny face flitting about his room all day. His ears drank in every tone of the cheery, bird-like voice, listening, at first, mechanically to its music; and then—his interest awakening—comprehending that it spoke to him of a world whose suffering—beside which his own paled and grew lighter to bear—was intensified by cruel poverty, by grinding want. His cheek burnt as he thought of his useless piles of gold, his rich lands and belongings, all of which would, by-and-by, go to the far-off cousin, the heir, whom he detested. How careful he had been all through his pampered life not to see the "stranger," that he might "take him in;" the "naked, whom he might have clothed;" and the "sick and in prison," whom he might have "visited." But now this little woman, whose hands had the magic of healing, dragged these suffering brothers and sisters out into the light.

"Never mind; it's not too late. I'll do some good before I die. Die! Why, I'm getting better every hour."

"You've worked a miracle, Nurse Eyre," said the jubilant doctor. "You have changed the wild beast into a meek lamb. Come, tell me your charm, and how you work it?"

"I?" Nurse Eyre's clear, child-like eyes grew round. "I have done nothing. I found Sir John frenzied with great sufferings, and very lonely, poor old man, so I tried to cheer him; that's all. I supposed he wanted a little love, a little sympathy, and I did what I could. It wasn't a mistake, was it?"

"A mistake!" The doctor looked down at her half amused, half puzzled. "Well, it was, and it was not. What you found was a selfish, tyrannical being—a great sufferer, I grant you. In your simple-hearted innocence you faced him as Una confronted the lion, and with a like result," and the doctor retreated to the window, gazing out silently, not upon the vista of swelling green turf and stately oaks, but upon a vision of a weary world of men and women, upon a "madness of the people" ever growing more frenzied for want of a few pure, trustful spirits to go in and out among them, pouring in oil and wine—that is love and sympathy—upon their wounds.

The once fiery Master of Chudleigh sits at the open window of a room facing right down the long straight avenue of stately trees by which all comers approach the house. Sir John still suffers—he always will—but his frenzy has gone; he bears his pains soldier-like, for has not a good woman's gentle voice made him acquainted with the "sorrowful sighing" of the suffering poor, thus, unconsciously to herself, setting a match to the spark of manly shame at his own mutinous fury? And the old man is lonely no longer—his heart is full of plans which his head and his hands execute. It is late in the day to begin doing good; but he is all the more eager to make up for lost time, and many a home, many a charity, is propped up by his far-reaching bounty.

And Nurse Eyre? Her work at Chudleigh is done for the present, and she has softly flitted out again into the world of suffering. Here and there—wherever, in fact, there is need for her gentle ministering—may cheery Nurse Eyre be found; beside the poor child dying, partly of starvation, partly of disease; and beside some wealthy loved one, to save whom showers of gold are vainly offered her could she but beat back the icy grasp of death. Treading in the Master's footsteps, she "goes about doing good."

### Notes from Australia.

(*By Our Own Correspondent.*)

(*Concluded from page lxxxvii*).

Now a word to medical students, who may perhaps fancy things are easier here than at home. A most amusing article has lately appeared in a daily paper entitled "A Modern Inquisition," and the account it gives of examinations ought to interest those in England who are at present debating this subject. The writer insists that the answer to the first question in the Shorter Catechism, "What is the chief end of man?" (or of woman, too, for that matter) should be amended so as to read, "To cram for matriculation, and to pass subsequent examinations." The day is not far distant when we shall read on the University notice-board, and subsequently in the columns of the daily press, an intimation something like the following :—

EXTRAORDINARY EXAMINATIONS.

22ND YEAR MEDICINE.

Nos. 1—10. Brain fever.
Nos. 11—20. Lunatic Asylum.
Nos. 21—25. Committed suicide.

The returns of the rest are incomplete.

The medical societies are now asking for fresh legislation on the subject of registering medical practitioners; this is only just, for why should our students pass such severe examinations if outsiders holding poor certificates can be put on the same register with them?

The Christmas appeals here were well responded to, and the Hospital Sunday Fund reached £12,482. This sum was divided amongst thirteen charities; Melbourne Hospital taking the giant's share of £3,600.

A singular case at the Women's Hospital has led to the censure of one of the medical staff, yet I cannot think the doctor was greatly to blame. A case of puerperal fever was brought to the hospital, and refused by Dr. Adam. Twenty-four hours after the woman was brought back again in a dying state, and admitted by Dr. Anderson to the septic cottage. Infectious cases are not taken at the Women's Hospital, and Dr. Anderson broke the rule; but many of us believe he would have been likewise censured had he turned the dying woman from the doors. When doctors disagree the wretched patient always suffers, and in this case the woman was left for more than 24 hours without seeing a doctor, they not caring to go near her, as it would prohibit them from attending ladies for several months. The woman would certainly have done better outside the hospital.

Christmas Eve here was wonderfully hot, the thermometer amusing itself variously between 90 degrees and 120 degrees. It is rather a sort of "duty pleasure" to turn out under that condition of things, but one gets used to it, and even extracts a good deal of enjoyment out of it. That afternoon I took a tramcar (you have none in London nearly equal to ours) up Elizabeth Street, and right on to the suburb of Brunswick. The streets were brilliant with well-dressed ladies, chiefly young, and our bonnie and happy-looking children were in force everywhere. The shops were gay, and verandahs and balconies were charming with tree-ferns. We passed the University with its many acres of "grounds," Trinity College, Ormond College (the gift of one munificent benfactor), and Prince's Park. Then come the smaller houses and shops correspondent, and half-way up a rather plain sort of street is the house I am in search of, the Brunswick Rescue Home. I know the sort of young women it is for, and am braced accordingly. I have been in places in England professedly dedicated to the same holy purposes, and found them dreary, messy, and prison-like. This Brunswick Home, worked in connexion with the Salvation Army, is unlike them. It does not belie its name. It looks like, and feels like, a "home." The religious side of things, however, is not forgotten. Over the entrance door appears the word "Immanuel," A dear, wise, strong, and mother-like woman presides over the Home, and has had wonderful success. She accepts her work as a God-given mission. Her good husband is an efficient coadjutor, combining authority with judgment and religious earnestness. The young women under their care—the care of Mr. and Mrs. Raske, had a better expression of face than I expected. The washing and ironing were going on as usual, and, in addition to these, some happy diligence was being shown in preparing the two largest rooms for a Home tea-party on Christmas Day. In only five years no fewer than 1,118 girls have passed through this institution, thirty being the average number of inmates for the year round. The regaining of sound physical health is accompanied with the regaining of an infinitely better moral tone. Not infrequently, I believe, it is the desire for this latter, rather than anxiety about the former, which leads to appli cations for admission. Notwithstanding all the æsthetic horrors of which the "Army" is guilty, it is impossible to deny that in many cases it accomplishes a good which others attempt only in vain.

### Medical Missionaries.

THE following interesting details have just come to us from Hankow, China, where two of the students from the Zenana Medical College have opened a medical mission in connection with the Wesleyan Missionary Society for Women and Children: "During the past twenty-five years, though not without some interruptions, medical mission work has been carried on in Hankow, but no medical mission exclusively for women and children was established until the year 1884, when this branch of Christian work was commenced by Mrs. North, of the Wesleyan Mission, who opened a little dispensary in Hankow, and during the few months she had charge of the work attended to some 2,000 patients. When once a beginning was made, the Ladies' Society in England took up the work, and appointed to the post a missionary lady, specially trained for this service; and for two years and a-half Miss Sugden has had charge of the work. During that period she has seen 13,117 out-patients (including 5,400 repeats), and has made 308 visits to the houses of patients unable to come to the dispensary, these being chiefly serious obstetrical cases. At first a room in the girls' day school was used as a dispensary; this was soon found to be inconveniently small, and a new room was erected; but as the work became more widely known, and the number of patients increased, and calls to serious cases outside multiplied, the need of further accommodation for in-patients became increasingly apparent. This need was stated to the committee of the Ladies' Society, who, with prompt generosity, collected upwards of £1,000 for the erection of a hospital, so that the present building is opened entirely free from debt. In this building, besides a dispensary, consulting, waiting, and nurses' rooms, there is one large ward capable of accommodating twelve in-patients; also four private wards and an operating theatre, etc. In the treatment of the more serious cases, Miss Sugden will have the assistance of the Rev. S. R. Hodge, M.R C.S., L.R.C.P., who has consented to act as consulting surgeon and physician. The surplus funds will provide instruments, furniture, etc., and thus only the working expenses be required from year to year. The Gospel will be daily preached to the out-patients in the waiting-room, and special attention given to the spiritual interests of the in-patients." Miss Sugden was trained at the Zenana Medical College, 58, St. George's Road, S.W.

### Everybody's Opinion.

[*Correspondence on all subjects is invited. No communications can be entertained if the name and address of the correspondent is not given, or unless one side of the paper only be written on.*]

### YORK HOME FOR NURSES.

DR. JAMES RAMSAY writes :—In your notice of this institution in the issue of your valuable journal I observe that by a printer's error the nurses are stated to have received as wages £82 6s. 9d. last year. This should have been £882 6s. 9d. Our report is in the hands of the printers, and we shall have pleasure in sending you a copy as soon as possible. Meantime, perhaps you will kindly correct the above error in your next number.

### BE COURTEOUS.

A. E. S. writes : "More harm is done by want of thought than by want of heart." A case in point came under observation the other day. A party of ladies, amongst them the wife of a high dignitary of the Church, were entertained by the house surgeon, at his own expense, at afternoon tea. They were then conducted over the hospital, where they gushed over the patients; particularly was the gushing evident in the nurses' wards! After seeing all they required to see they departed without even "Good afternoon" to the nurses in charge, or dropping sixpence into the hall box. Most likely this was simply want of thought, but the sooner such want of thought is brought to the notice of ladies the better both for the "classes and the masses." No doubt, counter charges of want of courtesy could be brought against sisters and nurses; if so, it is caused by the feeling amongst them, spite of improvements in hospitals and hospital manners, that they are looked upon as something below the general run of women. Expected by the public to be "angels in uniform," and do more work for less money than domestic servants; a good woman; the lady superintendent or nurse has an immense influence, and deserves all consideration and courtesy at the hands of visitors. Very few persons remember the words, "Be courteous" are as much a command as "Be sober."

### COMBINATION OF PRIVATE NURSES.

FAIRPLAY writes :—There seems to me a great deal of complaining from time to time from nurses belonging to institutions, and a growing desire for combination. I am a private nurse, but have not yet had need to join any institution. I think a system of co-operation might be found practicable. For instance, if a number of first-rate nurses would form a company and each give a sum of money to make a fund to begin business upon, say £20—then all earnings to be sent in and quarterly to be divided equally amongst them after expenses were cleared. With a few rules necessary for guidance they ought to work harmoniously. Starting in a neighbourhood where doctors abound they would be likely to get their patronage. Doctors, too, as a rule, are good friends to worthy nurses, and would not seek them so much from institutions if they were not the only convenient places to get them readily. If you think these suggestions worth insertion, I shall be pleased. Any nurses who may wish to fall in with such a plan I would be willing to correspond with.

[We have "Fairplay's" address, and will forward letters addressed to her at this office.—EDITOR.]

"X" WRITES :—Having read the letters which have appeared on the above subject in THE HOSPITAL, I am surprised that no one proposes to start a combination at once. I nursed for some years in connexion with a London nursing institution, against the management of which I have no special complaint to make, but I wished to be able to spend a few weeks at home occasionally. I also felt that I could not afford to give up so large a proportion of my earnings; so, at the end of my term, I withdrew from the institution and commenced work on my own account. I have suffered no inconvenience so far, having been one year with my present patient, but I should be willing to join any reasonable number of trained nurses in taking apartments, from which we could advertise, as papers are open to nurses as well as to institutions.

### GIVING AND TAKING.

"THE race of mankind would perish did they cease to aid each other. From the time that the mother binds the child's head till the moment that some kind assistant wipes the death-damp from the brow of the dying, we cannot exist without mutual help. All, therefore, that need aid have a right to ask it from their fellow-mortals; no one who holds the power of granting can refuse it without guilt."—SIR W. SCOTT.

### Appointments.

[It is requested that successful candidates will send a copy of their applications and testimonials, with date of election, to THE EDITOR, The Lodge, Porchester Square, W.]

THE HOSPITAL AT FIJI.—The appointment of matron-nurse to Fiji, about which there has been such excitement, is at last decided after a keen contest, and we are sure the right person has secured the post. The successful candidate, Miss M. A. Marshall, trained at Grimsby Hospital and Edinburgh Royal Infirmary. At the last-named place Miss Marshall carried off four first prizes at the annual examination, and this is scarcely to be wondered at, as at the age of 15 she took first-class honours in the Cambridge Junior Local Examination. Miss Marshall left Edinburgh to become matron of Warrington Infirmary, and she has had temporary charge of Teignmouth Infirmary and the Cambridge Home. For the last three years Miss Marshall has acted as head lady nurse of Wonford House, Exeter, and her departure to such a far country will be a loss to many of her friends. We hope Miss Marshall will follow Miss Blake's example, and send us an account of her experiences in Fiji.

BIRMINGHAM EYE HOSPITAL.—At a meeting of the committee of this institution, held on February 28th, Miss Susan E. Vaux, trained at the Middlesex, was appointed matron of this hospital.

### Vacancies.

The following vacancies are announced :—

### Matrons.

Princess Alice Memorial Hospital, Eastbourne; £50.

Weymouth Royal Hospital, £35.

### Nurses.

Nurses' Home, Truro (monthly); £25.

Temperance Hospital, Hampstead (night); £25.

Southport Trained Nurses' Home.

Eston Hospital; £25.

Devon Hospital, Plymouth, £25.

Hertford Infirmary; £20.

North London Institution, Finsbury.

Royal United Hospital, Bath; £26.

Portsmouth Royal Hospital; £25.

Eastbourne Nursing Institution.

Bowdon Hospital for Consumption (night).

Kingston Nurses' Home, Hull.

Levick Paris Institution; several.

Brecon Infirmary; £18.

Royal Surrey County Hospital, Guildford; £18.

Barnhill Hospital, Glasgow; £24.

Bolton Infirmary and Dispensary.

Southport Infirmary; £20.

Bradford Infirmary; £25.

Nottingham Nursing Institution; £25.

City Nursing Institution, Worcester.

St. John's Institution, Southport.

Rous Hospital, Newmarket; £20.

Stroud Hospital (night); £20.

West Bromwich District Hospital; £22.

Dartmouth College Hospital; £35.

Nursing Institution, 96, 97, 98*a*, Wimpole Street, London, W., Mr. Wilson has several vacancies for thoroughly-trained Nurses.

Stroud Hospital; £20.

Southport Trained Nurses' Home; several.

Church Deaconesses' House, Chester; £24; several.

Darlington Fever Hospital (head); £26.

### District Nurses.

Holloway Road Home.

Bolton.

Camborne, Cornwall.

Cheltenham District Nursing Association; £22.

### Probationers.

Batley Cottage Hospital, Yorks.

Hammerwich Cottage Hospital.

Cromer Cottage Hospital.

Bolton Infirmary and Dispensary.

Durham County Hospital.

Halifax Borough Hospital; £14.

Boscombe Cottage Hospital, Bournemouth.

### Notes and Queries.

TO CORRESPONDENTS.—1. Questions may be written on post-cards. 2. Advertisements in disguise are inadmissible. 3. In answering a query please quote the number. 4. If a private answer is desired, a stamped addressed envelope must be forwarded.

### Queries.

(123) *Naval Nurses.*—Where can I get information as to the Navy nurses, and what training must they have before entering?—*A. R.*

(124) *Home Wanted.*—Can any one tell of a home where a poor girl suffering from epilepsy can be received permanently?—*Edith S.*

(125) *Air Cushion.*—Will anyone lend or give an air cushion to a poor person?—*Miss Wilson*, 45, Colville Gardens, W.

(126) *Training in Midwifery.*—Can anyone tell me of a hospital where a nurse can receive training in midwifery if she gives her services for three months?—*Nurse B.*

### Answers.

(121) *African Nursing.*—Particulars about nursing prospects at Cape Town could be obtained by writing to the Sister Superior, New Somerset Hospital, Cape Town. There is great demand for private nurses, as the institutions and hospitals are for nursing the poor, and they will only send out nurses as a great favour.—"*Pig-girl.*"

